# Clinical significance of premature ventricular contraction among adult patients: protocol for a scoping review

**DOI:** 10.1186/s13643-019-1168-4

**Published:** 2019-11-01

**Authors:** Sukardi Suba, Michele M. Pelter

**Affiliations:** ECG Monitoring Research Laboratory, Departement of Physiological Nursing, University of California, San Francisco (UCSF), 2 Koret Way, N631, San Francisco, CA 94143-0610 USA

**Keywords:** Scoping review, Premature ventricular contraction, Clinical significance, ECG monitoring

## Abstract

**Background:**

Premature ventricular contractions (PVCs) are one of the most common arrhythmias detected from electrocardiographic (ECG) monitoring. PVCs were thought to cause lethal arrhythmias and thus were closely monitored and treated. However, in current practice, PVCs generally do not required treatment. There is also concern that PVCs contribute to excessive alarms and lead to alarm fatigue. Practice guidelines for in-hospital monitoring state that monitoring for PVCs may be indicated on some patients but do not recommend continuous ECG monitoring. Despite these recommendations, PVC monitoring practices remain part of routine care, especially in the intensive care unit, for worry of missing potentially significant arrhythmia events. A thorough scoping review of the literature regarding the clinical significance of PVC is imperative, precisely to map out the evidence on the diagnostic and prognostic values of PVCs and to identify research gaps on this issue.

**Methods:**

The primary question of this review is “what is the clinical significance of PVCs in adults?” Preparation of this scoping review will use the PRISMA-P statement. A scoping review framework by Arksey and O’Malley will be adopted. In identifying relevant studies, the Population-Concept-Context (PCC) framework by the Joanna Briggs Institute will be used. A search strategy will be developed, and four major electronic databases will be searched: CINAHL, Embase, PubMed, and Web of Science Core Collection. Manual searches will also be conducted. The study selection process will adopt the 2009 PRISMA flow diagram. EndNote X8 will be used to manage citations, as well as for duplicates screening in addition to Microsoft Excel 2016. Two independent reviewers will assess potential studies in detail against inclusion criteria. A standardized data extraction form will be developed. Finally, critical appraisal will be conducted using a tool adapted from the Quality Appraisal Checklist by the National Institute for Health Care Excellence (NICE).

**Discussion:**

We believe this scoping review will provide a general foundation of evidence on the potential significance of PVCs concerning its diagnostic and prognostic value among the adult patient population. The findings will allow us to map out research gaps on this topic that could shape future research and ultimately clinical practice.

**Scoping review registration:**

This scoping review has been registered in the Open Science Framework (OSF), DOI: https://doi.org/10.17605/OSF.IO/GAVT2.

## Background

Alarm fatigue occurs when clinicians are desensitized by excessive numbers of alarms. As a result, the alarm sounds become “white noise” that is perceived as part of the typical working environment in the intensive care unit (ICU). Consequently, alarms might be silenced without checking the patient, adjusted by turning the volume down, or in extreme cases permanently disabling to minimize the burden to the nurse and patient. These responses have been recognized as a significant patient safety hazard leading to unintended consequences for patients, including death, due to missed true alarms [1]. The Association for the Advancement of Medical Instrumentation (AAMI) and the U.S. Food & Drug Administration (FDA) have warned of deaths due to alarm silencing on patient monitoring devices [2]. Likewise, several other agencies and national organizations, such as the Emergency Care Research Institute (ECRI) and The Joint Commission, have issued alerts about alarm fatigue being a major patient safety concern [3, 4]. The American Association of Critical-Care Nurses (AACN) recently published a practice alert as an evidence-based tool to guide both nurse clinicians and leaders in reducing false or nonactionable alarms with the goal of preventing alarm fatigue [5].

In clinical settings, one of the most common rhythms detected from electrocardiographic (ECG) monitoring in the ICU are premature ventricular contractions or PVCs [1]. When ECG monitoring was first introduced in the late 1960s, PVCs were thought to cause lethal arrhythmias (i.e., ventricular tachycardia [VT] and/or ventricular fibrillation [VF]); hence, PVCs were carefully monitored for and treated [6]. However, such practices changed when in the late 1980s, the Cardiac Arrhythmia Suppression Trial (CAST) showed that treatment for PVCs with antiarrhythmic drugs was associated with more deaths compared to placebo [7]. As a result of this important study, PVCs became considered a nonactionable arrhythmia alarm [1].

Despite being considered nonactionable, PVC alarms in many institutions are still turned on (audible or inaudible) during continuous ECG monitoring. In a recently published study, Drew and colleagues found 2,558,760 alarms were generated from bedside monitors [1]. Of the total number of alarms, 854,901 (33%) were for PVCs. While the PVC alarms were configured as in-audible text messages during this study, we believe that flashing warnings on the bedside monitor, while inaudible, still distract nurses and lead to alarm fatigue. Simpson and Lyndon, in their recently published study, described how neonatal ICU nurses “…hate seeing flashing…” (p. 4), with regard to inaudible text messages as they felt that there was something wrong with the patient [8]. This study illustrates that not only audible but also inaudible text alarm significantly contributes to alarm fatigue.

In the recently updated American Heart Association (AHA) Practice Standards for ECG Monitoring in Hospital Setting, recommendation for PVC monitoring states “PVCs and nonsustained VT are not immediately life-threatening, and in the absence of other indications for monitoring in hospitalized patients, continued arrhythmia monitoring may be considered but is not required” (p. e300) [9]. However, benefit or usefulness of this recommendation in clinical practice is less well-established (class IIb), and this practice is mainly based on expert opinion or standard of care (level of evidence: C) [9]. Considering the high volume of nonactionable PVC alarm, it is fair to say that PVC alarms contribute to alarm fatigue. Thus, turning off PVC monitoring completely from the bedside monitor seems to be reasonable since it will reduce a large number of nonactionable alarms. In reality, however, clinicians generally feel uncomfortable about the idea of removing/disabling PVC monitoring from routine continuous ECG monitoring. For example, there is a concern of potentially missing patients who are at high risk for developing torsade de pointes. In these patients, bradycardia followed by PVCs following a compensatory pause are a marker for TdP [1]. PVCs can also occur in patients with electrolyte disturbances, such as hypokalaemia and magnesium deficiency [9]. Unfortunately, there is no concrete evidence to support either approach (i.e., the need to monitor or turn off).

Because PVC monitoring practices and recommendations are uncertain, a thorough review of the literature regarding the clinical significance of PVCs is imperative. Furthermore, it is evident that there is a critical conflict between establishing evidence-based practice and the empirical evidence in this area. Assessing what is known and unknown on this topic may provide a more precise understanding of how to interpret the clinical significance of PVCs in general and how PVCs may differ between patients in the outpatient and inpatient settings. A preliminary search of the literature of major databases (i.e., PROSPERO, the Cochrane Library, and the JBI Database of Systematic Reviews and Implementation Reports) indicated that there are no current or underway systematic reviews on this topic. Ongoing reviews related to PVCs in the PROSPERO registry focus on predictive factors of ejection fraction after cardiac ablation intervention and PVC ablation among children. Therefore, in this review, we seek to describe what it means clinically when a patient has PVCs. Specifically, this scoping review will examine the clinical significance of PVCs in terms of their potential prognostic and diagnostic importance among adult patients in both outpatient and inpatient settings.

## Methods

This protocol has been registered in the Open Science Framework (OSF), DOI: https://doi.org/10.17605/OSF.IO/GAVT2. In preparing this protocol manuscript, the Preferred Reporting Items for Systematic Reviews and Meta-Analyses Protocols (PRISMA-P) statement [10] was used, as well as the PRISMA-P Checklist (Additional file 1). This proposed scoping review will adopt the scoping review framework by Arksey and O’Malley [11], which involves the following stages:

Stage 1: Identifying the research question

Stage 2: Identifying relevant studies

Stage 3: Study selection

Stage 4: Charting the data

Stage 5: Collating, summarizing and reporting the results

In parallel, we will also adapt the scoping review protocol outlined by The Joanna Briggs Institute (JBI) [12].

### Stage 1: Identifying the research question

The main research question is, “what is the clinical significance of PVCs in adults?” The research sub-questions are:

1. “What is the prognostic value, if any, of PVCs among the adult population?”
2. “What is the diagnostic value, if any, of PVCs among the adult population?”

### Stage 2: Identifying relevant studies

In this stage, the JBI protocol provides clearer guidance and, thus, will be adapted. The search strategy is aimed at locating published studies. Following the Methodology for JBI Scoping Review [12], we conducted a limited search of PubMed and Embase to identify any subject headings on this topic. After an initial search, it was determined a broader search strategy was needed to ensure capturing as many relevant studies as possible (Table 1). We will search four major electronic databases, which include CINAHL, Embase, PubMed, and the Web of Science Core Collection. In addition, manual searches will also be conducted by reviewing our personal libraries and the reference lists of all studies selected for critical appraisal. Unpublished studies and grey literature will not be included.

**Table 1:** Search strategy

Database	Query
CINAHL	(MH “Premature Ventricular Contractions”) *Filters: Abstract Available, Academic Journals, English.*
Embase	(‘heart ventricle extrasystole’/exp. OR ‘heart ventricle extrasystole’) AND ([article]/lim OR [article in press]/lim) AND [english]/lim AND ([young adult]/lim OR [adult]/lim OR [middle aged]/lim OR [aged]/lim OR [very elderly]/lim) AND [clinical study]/lim
PubMed	premature ventricular contraction [MeSH] *Filters: Humans, English.*
Web of Science Core Collection	((premature ventricular contraction) OR (ventricular extrasystole) OR (ventricular extra systole) OR (premature ventricular beat)) *Filters: Document types (Articles), English.*

In identifying relevant studies for this review, we used the Population, Concept, and Context (PCC) framework by the JBI for scoping review [12]. The PCC framework aligns with our purpose to scope a broader literature, following our search strategy. Therefore, our inclusion criteria should meet the PCC as follows:

#### Participants

This review will include studies that focus on the adult patient population aged 19 years or older, identified as having PVCs. Studies on pregnant women and athletes will be excluded, considering the pathophysiological mechanism would be different from the general adult patient population. Studies on exercise-induced PVC will be included if the procedure was performed in a clinical setting as part of a screening or diagnostic test.

#### Concept

The concept of interest for this scoping review is the clinical significance of PVCs, which is comprised of prognostic and diagnostic value. For prognostic value, we specifically are interested in assessing the predictive value of PVCs on patient outcomes (e.g., rate/risk for mortality, hospital admission, emergency room visit, hospital re-/admission, and hospital or ICU length of stay). For the diagnostic value, we will focus on identification or determination of specific disease or “the distinguishing of one disease or condition from another” (Medical Subject Headings [MeSH] PubMed; *diagnostic value*).

#### Context

The context of the present review is both outpatient and inpatient settings. There is no limitation in terms of the geographical origin of the study, racial background, and gender of participants.

This scoping review will only consider primary experimental, observational, and descriptive studies with quantitative data. Therefore, individual case reports, systematic reviews, meta-analysis, and qualitative studies will be excluded. For this review, we will also exclude studies that combine PVCs with other types of arrhythmias (e.g., PVC and atrial premature complex [APC] are combined as one variable of “premature complexes”). Studies that focus on specific ECG features of PVC (e.g., duration of the QRS complex, coupling interval duration, and PVC patterns) will be included only if the studies correlate such features with patient outcomes, hence providing data on clinical significance of the PVCs. Only studies published in English will be included, and no restrictions on the publication date of the studies.

### Stage 3: Study selection

**Fig. 1:**
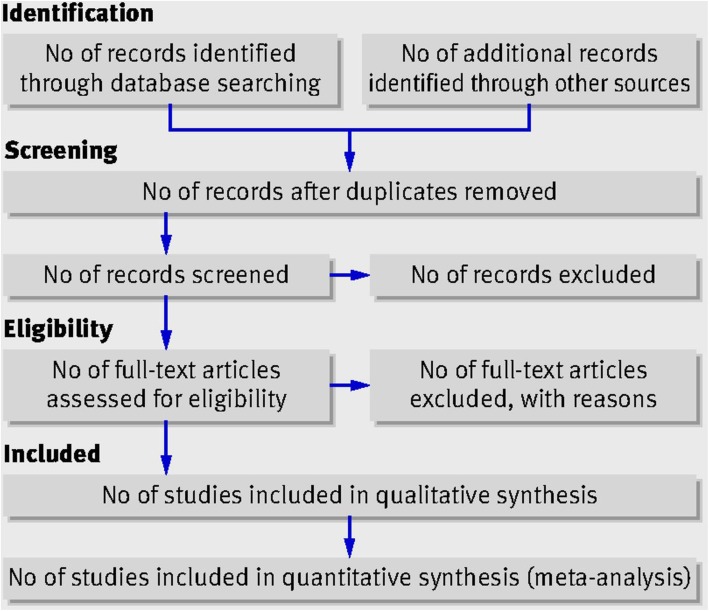
Preferred Reporting Items for Systematic Reviews and Meta-Analyses (PRISMA) flow diagram [13]

The study selection process will adopt the Preferred Reporting Items for Systematic Reviews and Meta-Analyses (PRISMA) flow diagram (Fig. 1) [13]. Following the search, all identified citations will be collected and uploaded into EndNote X8 (Clarivate Analytics, PA, USA) to remove duplicates. The remaining citations will then be exported to Microsoft Excel 2016 (Microsoft, Redmond, WA) for additional screening for duplicates. After removing all duplicates, titles and abstracts the articles will be screened against the inclusion and exclusion criteria. The full text and citation of potential studies will be retrieved and imported into EndNote X8. Potential articles will be assessed in detail against the inclusion criteria by two independent reviewers (SS and MMP). Any disagreements that arise between the reviewers will be resolved through discussion. When deemed necessary, a content expert (cardiologist) will be invited as a third reviewer to make the ultimate decision. Reasons for excluded studies will be recorded and reported.

### Stage 4: Charting the data

We will use a data extraction form (Table 2) to ensure standardized data retrieval from the included studies. The data extracted will include specific details about the population, concept, context, study methods, and critical findings relevant to the review objective. The data extraction form will be modified and revised if necessary, during the process of extracting data from each included study. Modifications will be reported in detail in the full scoping review report. Where required, we will contact the authors of included studies to request missing or additional data.

**Table 2:** Data extraction form

Author and year	
Title of the study	
Study aim/research question	
Population	
Country	
Sample size	
Patients’ characteristics (age, gender)	
Patients with/without cardiovascular disease	
Primary/secondary end point	
Intervention (if any)	
Study design	
Setting (ambulatory/outpatient, emergency department, in-hospital)	
Data collection/methodology	
ECG data collection procedure (duration, electrode lead placement)	
ECG recordings method (standard 12-lead, EASI leads, Holter, bedside monitor)	
ECG data annotation method (if applicable)	
PVC criteria	
Key findings	
Conclusion of the study	
Comments	

### Stage 5: Collating, summarizing, and reporting the results

The extracted data will be presented in either diagrammatic or tabular forms that align with the objectives of the scoping review. Based on the data extracted and charted in stage 4, we will conduct a descriptive analysis and present the narrative results in terms of numerical data presented in the included studies (e.g., statistical data) [11]. Also, evidence of the literature will be organized based on certain aspects that are a priority to address the objectives of this scoping review, to allow us to map research gaps in this area [11]. These aspects include, but not limited to, age range, gender, and clinical settings (e.g., ambulatory clinic, emergency department, and in-hospital setting), study design, data collection procedure, patient population (e.g., patients with or without cardiac disease), outcomes, and the clinical significance of PVCs in terms of their prognostic and diagnostic value. Such information will be summarized in a table, along with essential characteristics of all the included studies, additional commentary section, and knowledge gaps between studies.

#### Critical appraisal

Assessment for risk of bias is not required for a scoping review. However, we plan to conduct critical appraisal of included studies using a tool (Table 3) adapted from the Quality Appraisal Checklist—Quantitative Studies Reporting Correlations and Associations by the National Institute for Health Care Excellence (NICE) [14]. This process can help us identify good quality studies for our future work on a similar topic. The NICE tool consists of five major items: study population and participants, selection and methods, outcomes, analysis, and summary. One item, “is the setting applicable to the UK” was removed because it was irrelevant for this scoping review.

**Table 3:** Quality Appraisal Checklist—Quantitative Studies Reporting Correlations and Associations [14]

Population	
Source of population well described	
Eligible population representative of the source of population	
Participants represent eligible population	
Selection/methods	
Selection bias minimized	
Reasonable variables selection	
Acceptably low contamination	
Confounding factors identified and controlled	
Outcomes	
Outcome measures and procedures reliable	
Outcome measurements complete	
All important outcomes assessed	
Similar follow-up time in exposed and comparison groups	
Follow-up time meaningful	
Analyses	
Study was sufficiently powered to detect an intervention (if any)	
Multiple explanatory variables considered in the analysis	
Analytical methods appropriate	
Precision of association given or calculable and meaningful	
Summary	
Study results internally valid	
Findings are generalizable to the source population	

## Discussion

We believe this scoping review will provide foundational knowledge and evidence on the potential clinical significance of PVC among the adult patient population. Interestingly, since the 1989 CAST study report [7], PVCs are generally viewed as a low priority arrhythmia and do not require treatment. However, in a recent meta-analysis by Ataklte and colleagues [15], they found a significantly increased risk for sudden cardiac death and total cardiac death among the general population who had frequent PVCs (i.e., at least one PVC on a 2-min ECG recording). We suspect that there may be differences in evidence on the significance of PVCs between the general population without cardiac disease to patients with cardiac disease and to patients who are hospitalized. Mapping out the evidence that exists on the significance of PVCs on patient care in different settings will provide insights on the knowledge gaps and potentially inform future research in this area. In addition, this scoping review will also inform our current project in assessing the utilization of continuous ECG monitoring for PVC among the adult patient population in the ICU. By knowing the current state of the evidence on the clinical significance of PVC, we will have a better direction in assessing the importance and relevancy of PVC monitoring, how it may impact patient care and patient outcomes, and how to better monitor for PVCs without overburdening clinicians with excessive alarms. Finally, to our knowledge, scoping review is the best approach to address the uncertainty of evidence in this topic, considering that this method allows us to conduct an in-depth exploration of the existing evidence.

## Supplementary information

**Additional file 1.** PRISMA-P 2015 Checklist: Clinical significance of premature ventricular contraction among adult patients: protocol for a scoping review.

## Data Availability

Not applicable.

## Abbreviations

AACN: American Association of Critical-Care Nurses
AAMI: Association for the Advancement of Medical Instrumentation
AHA: American Heart Association
CAST: Cardiac Arrhythmia Suppression Trial
ECG: Electrocardiographic
ECRI: Emergency Care Research Institute
FDA: Food & Drug Administration
ICU: Intensive care unit
JBI: Joanna Briggs Institute
NICE: National Institute for Health Care Excellence
PCC: Population, Concept, and Context
PRISMA: Preferred Reporting Items for Systematic Reviews and Meta-Analyses
PROSPERO: International Prospective Register of Systematic Reviews
PVC: Premature ventricular contraction
UK: United Kingdom
US: United States
VF: Ventricular fibrillation
VT: Ventricular tachycardia
